# Supplementary material for: Structural basis for specific inhibition of the highly sensitive ShHTL7 receptor
Source: EMBO Rep. 2018 Jul 18;19(9):e45619. doi: 10.15252/embr.201745619 (PMC6123649; doi:10.15252/embr.201745619)
Supplement: Supplementary file 1 — Appendix [file EMBR-19-e45619-s001.pdf]

Appendix

Structural basis for specific inhibition of the highly sensitive ShHTL7 receptor

Authors

Umar S. Hameed[1,‡], Imran Haider[2,‡], Muhammad Jamil[2], Boubacar A. Kountche[2], Xianrong Guo[3], Randa A. Zarban[2], Dongjin Kim[2], Salim Al-Babili[2,*], Stefan T. Arold[1,*]

Appendix - Table of Contents

Appendix Figure S1, Page 2

Appendix Figure S2, Page 3

Appendix Figure S3, Page 4

Appendix Figure S4, Page 5

Appendix Figure S5, Page 6

Appendix Figure S6, Page 7

Appendix Figure S7, Page 8

Appendix Figure S8, Page 9

Appendix Table S1, Page 10

Appendix Text S1, Page 10-11

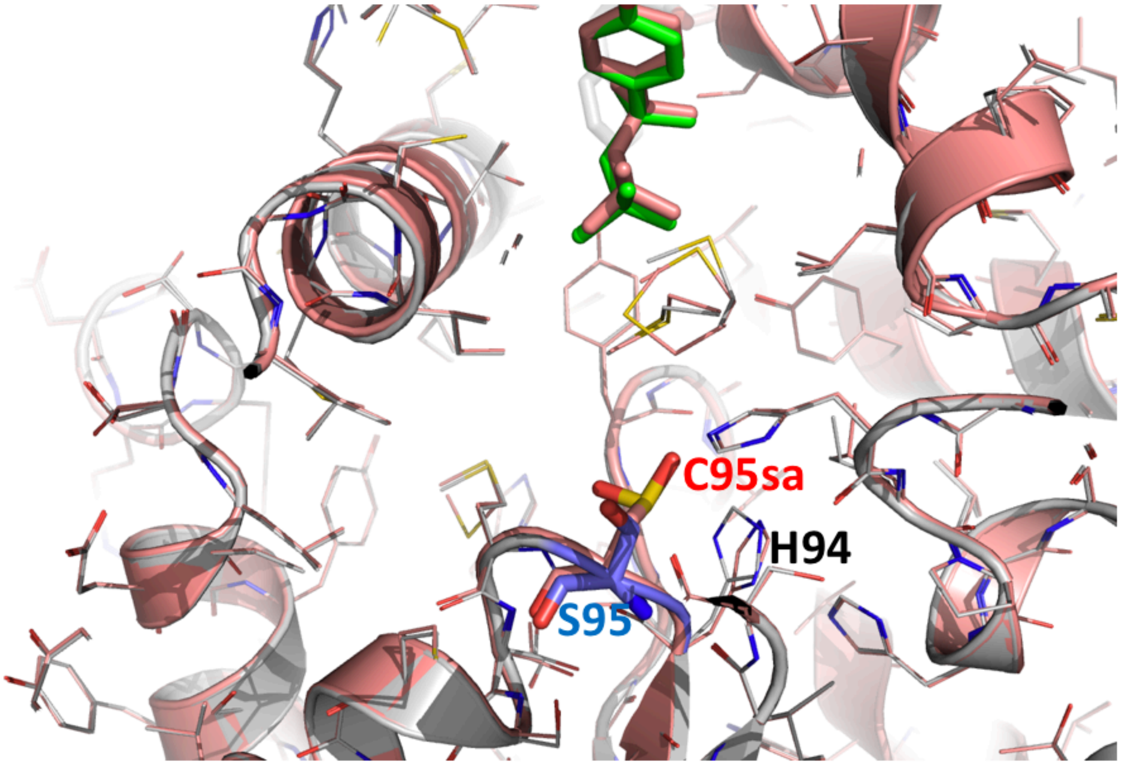

Appendix Figure S1. Structural effects of mutations on ShHTL7.

Superimposition of ShHTL7 wt onto the S95C mutant. Zoom into the active side pocket. ShHTL7: grey; bound Triton: green. ShHTL7$_{S95C}$: salmon; bound Triton: salmon. C95sa: Cysteine 95 modified into sulfinic acid. RMSD of 0.11 Å.

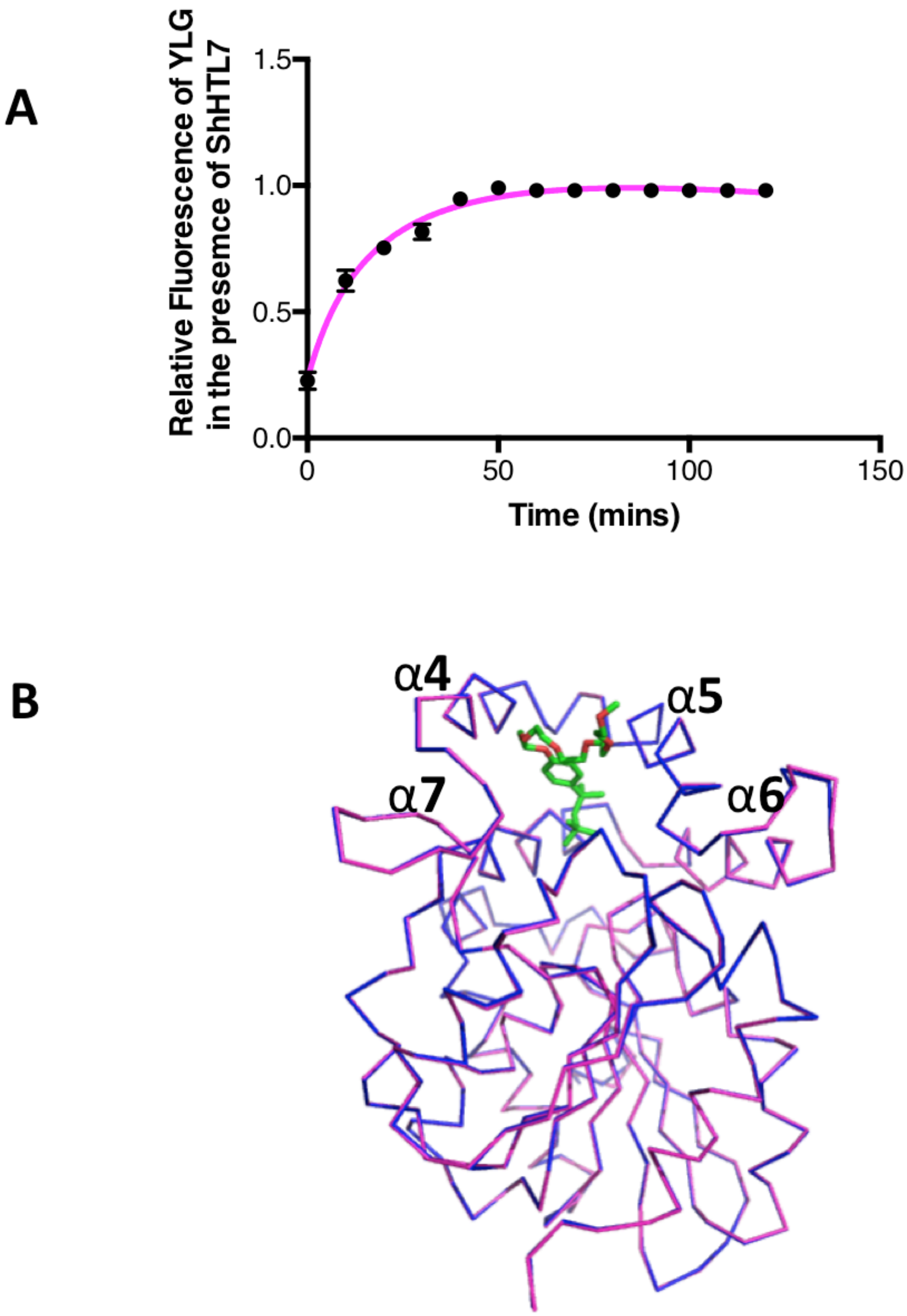

Appendix Figure S2. YLG hydrolysis by ShHTL7 and triton bound structure of ShHTL7.
A, YLG hydrolysis by ShHTL7 at different time points and each point corresponds to 10 mins interval. Data are mean ± S.D, $n = 3$.

B, Apo-ShHTL7 from the P65 space group (blue), superimposed onto the Triton-bound ShHTL7 crystallized in P65 (purple) (r.m.s.d. of 0.11 Å).

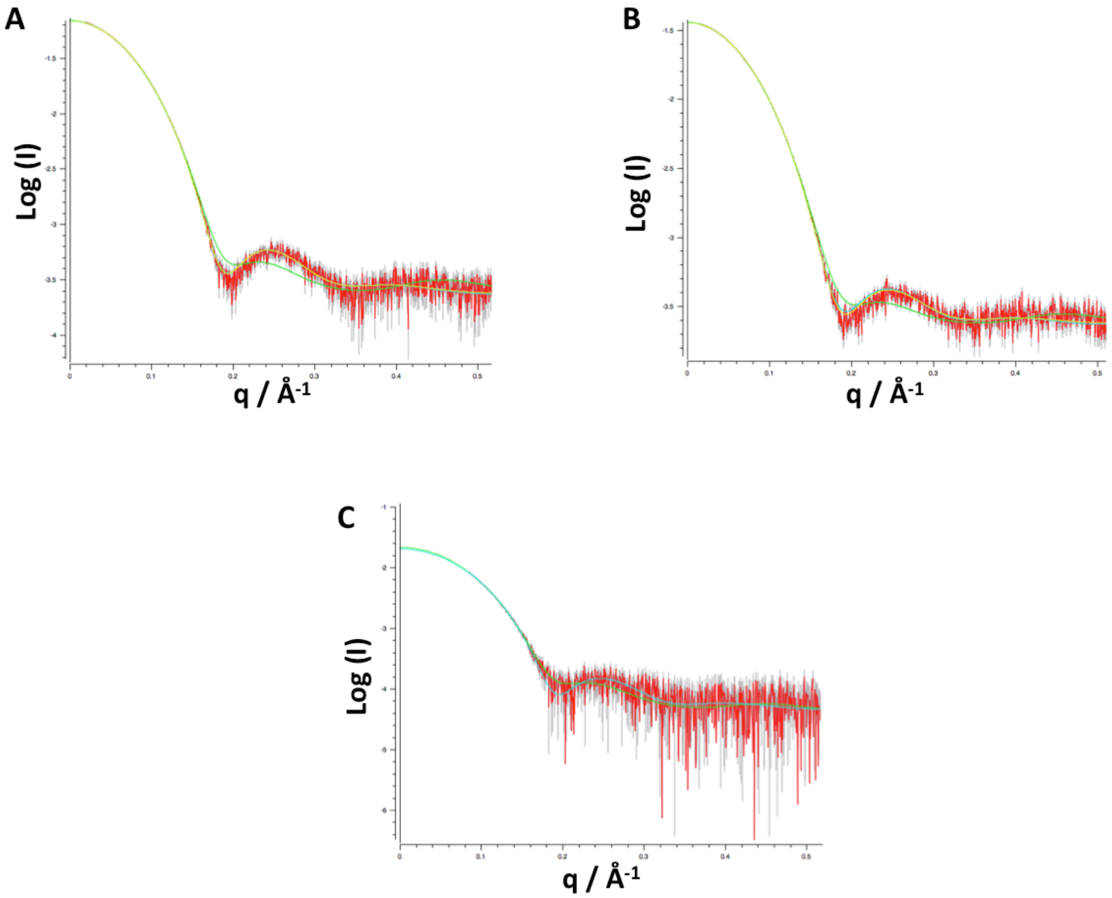

Appendix Figure S3. Structural effects of ligands on ShHTL7.

A, SAXS scattering data for apo-ShHLT7 (red lines) superimposed onto the fitted model–derived SAXS data for apo-ShHLT7 (I222 form; yellow line), and a homology model of ShHLT7 in the structure it would adapt when bound to ShMAX (green line). This homology model was built based on PDB 5hzg and on our apo-ShHTL7 structural data.

B, SAXS scattering data on Triton-bound ShHLT7 (red lines) superimposed onto the fitted model–derived SAXS data for apo-ShHLT7 (I222 form; yellow line), Triton-bound ShHTL7 (P65 form; cyan blue line) and the modelled ShMAX2-bound form of ShHTL7 (green line).

C, SAXS scattering data on GR24-bound ShHLT7 (red lines) superimposed onto the fitted model–derived SAXS data for apo-ShHLT7 (I222 form; cyan blue line), and the modelled ShMAX2-bound form of ShHTL7 (green line).

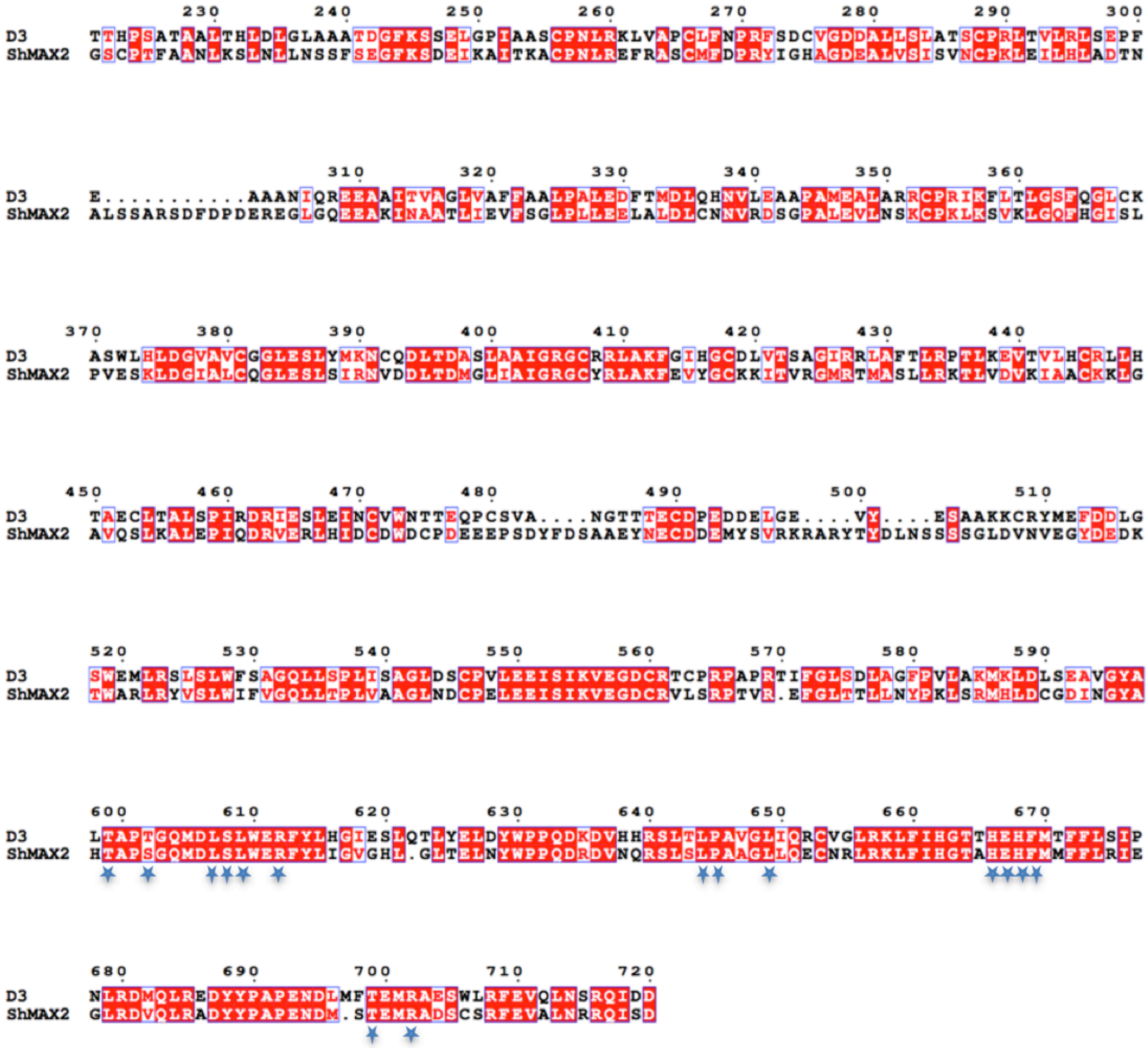

Appendix Figure S4. Sequence alignment of ShMAX2 and D3 (Rice MAX2). Red background: strict identity; Red letter: similar physiological character; blue box: highly conserved blocks. Residue marked (★) are involved in binding to ShHTL7.

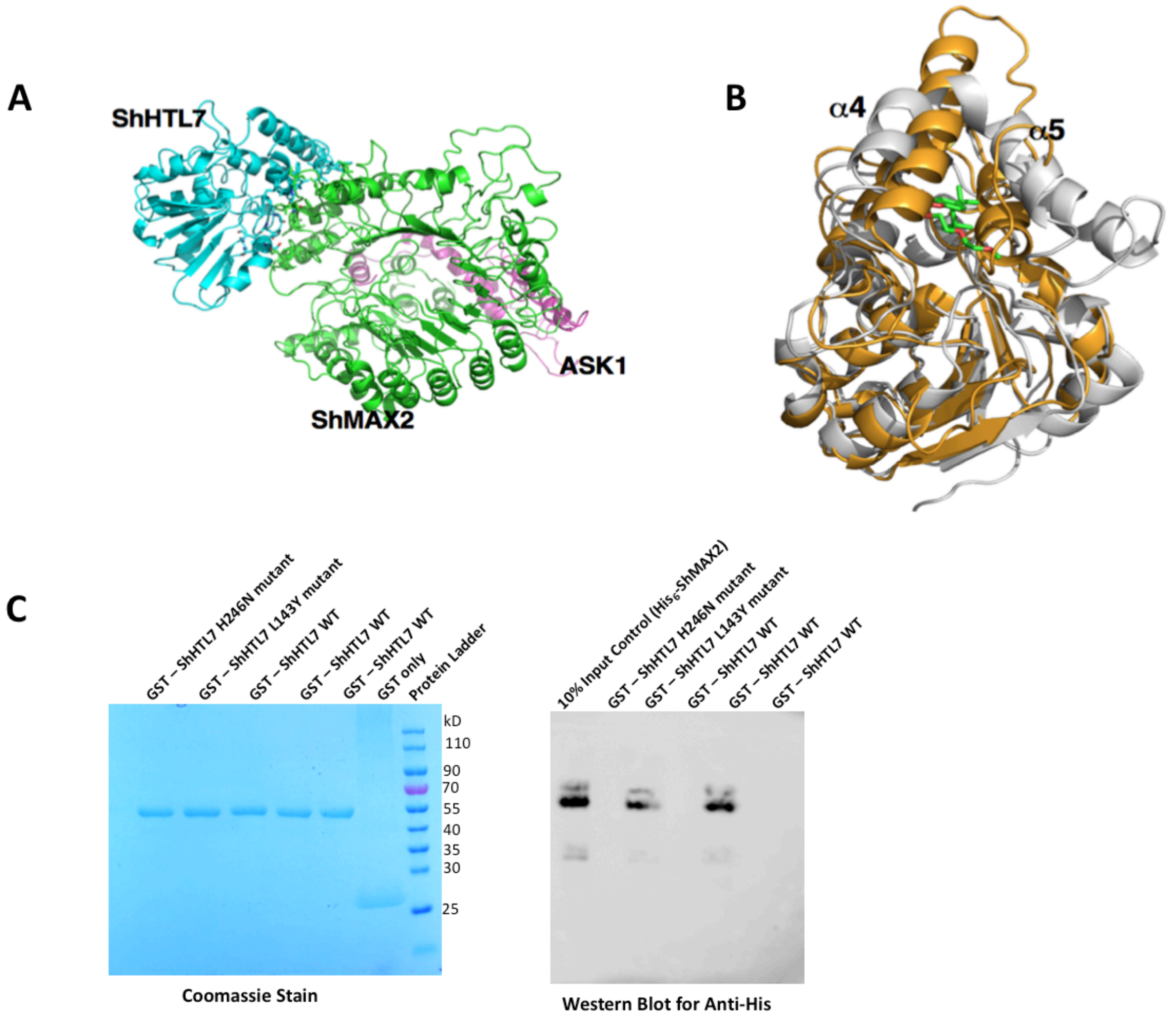

Appendix Figure S5. Interaction between ShHTL7 and ShMAX2.

A, 3D homology modelled structure of ShHTL7 (cyan), ShMAX2 (green), ASK1 (pink) complex using the template pdb: 5hzg.

B, ShHTL7-Triton bound structure (grey) superposed over modelled ShHTL7 bound to MAX2 (pale orange) with RMSD of 1.11 Å.

C, Full images of cropped picture in GST pulldown of ShMAX2 by ShHTL7 and its mutants shown in Fig 4A.

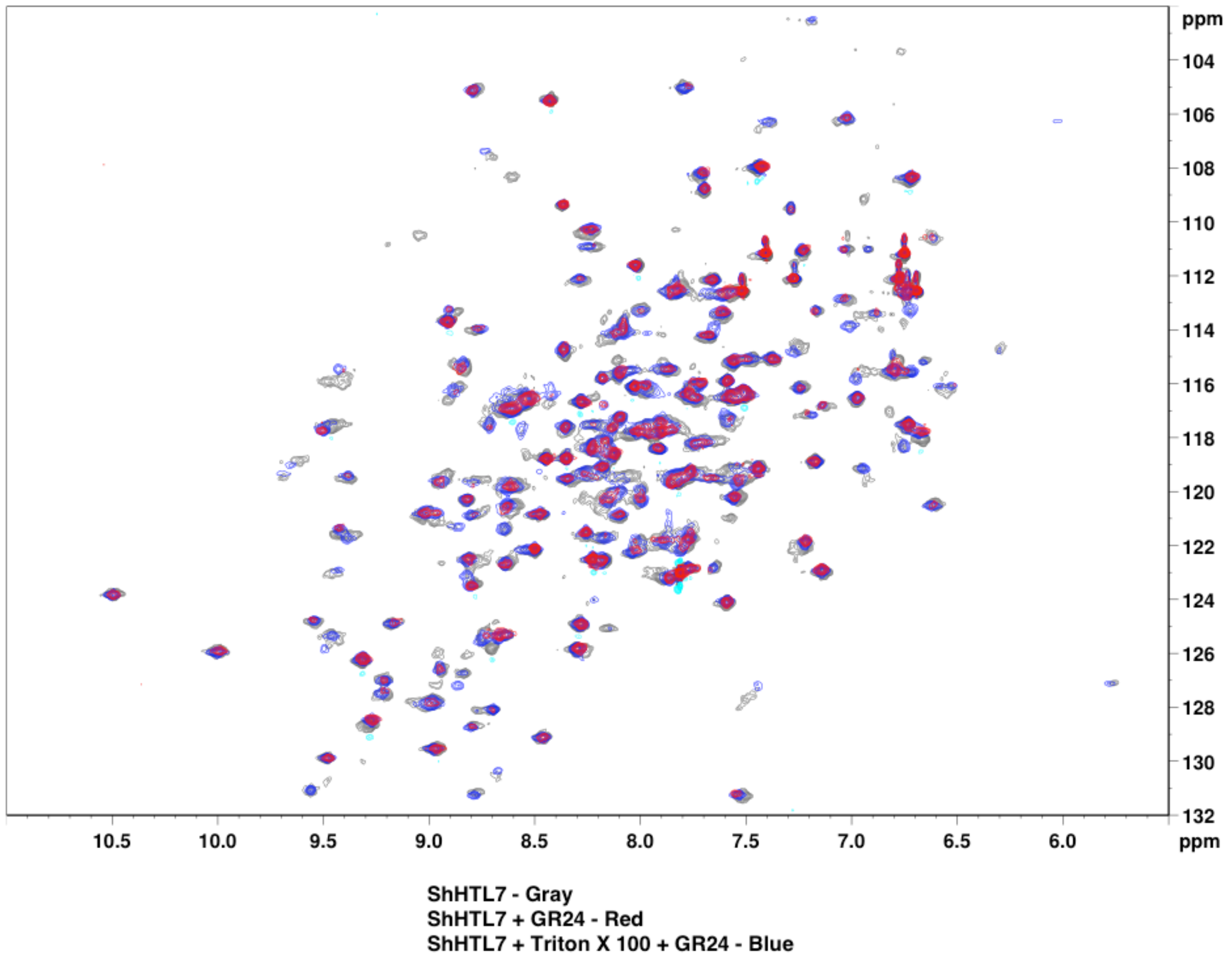

Appendix Figure S6. Triton blocks structural changes induced by GR24 in ShHTL7.

^{15}N HSQC spectrum of 0.3 mM ^{15}N-apo-ShHTL7 (gray) superimposed on the ^{15}N HSQC spectra of 0.3 mM ^{15}N-ShHTL7 (red) in presence of GR24 at the final ShHTL7–GR24 ratio of 1:1.5, and on the of 0.3 mM ^{15}N-ShHTL7 purified with Triton, in presence of GR24 at the final ShHTL7–GR24 ratio of 1:1.5 (dark blue).

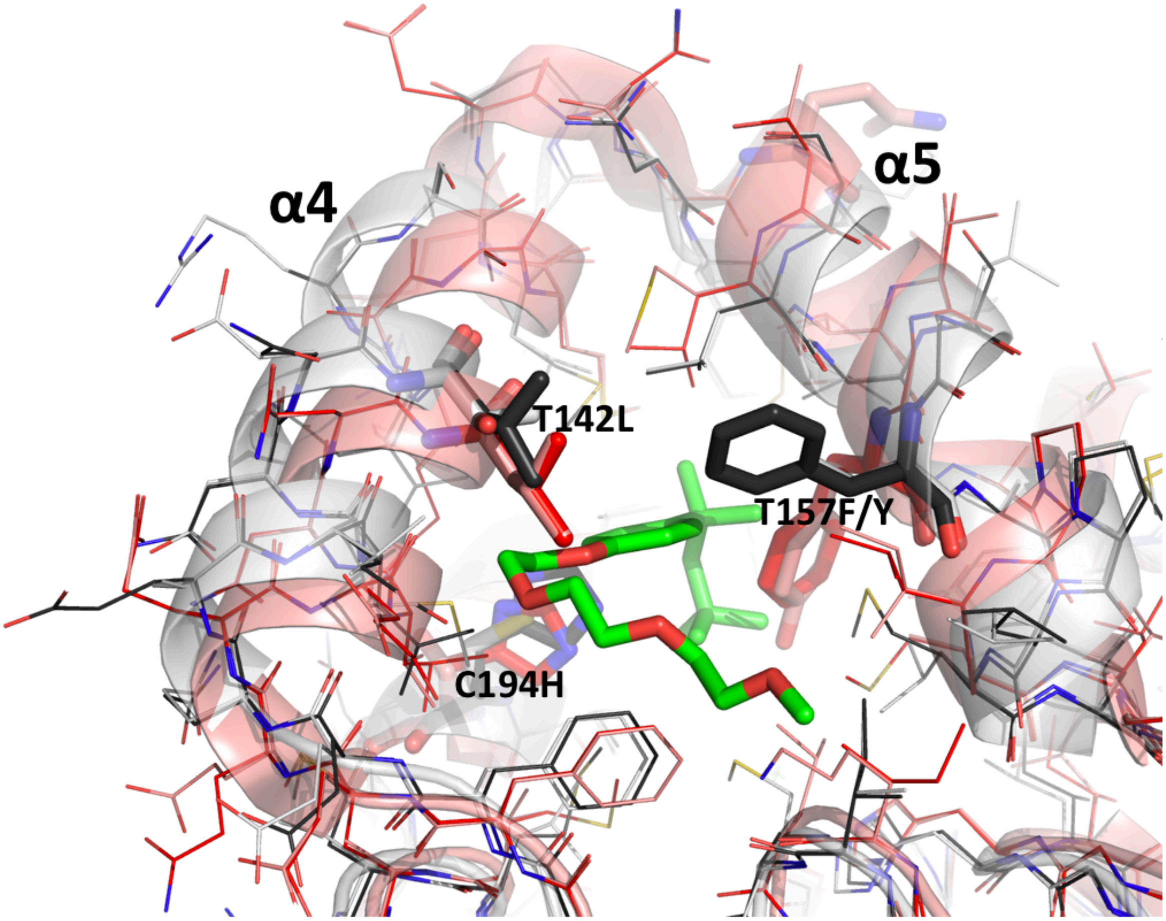

Appendix Figure S7: Evaluation of the Triton-binding capacity of ShHTL5 and ShHTL6. Superimposition of the crystal structures of ShHTL5 (PDB id 5cbk; light red) and ShHTL7 (light gray) bound to Triton (green carbons, red oxygens). The homology models of ShHTL6 are shown in dark gray (produced based on ShHTL7 as a template) and red (based on ShHTL5). RMSD of 0.743 Å.

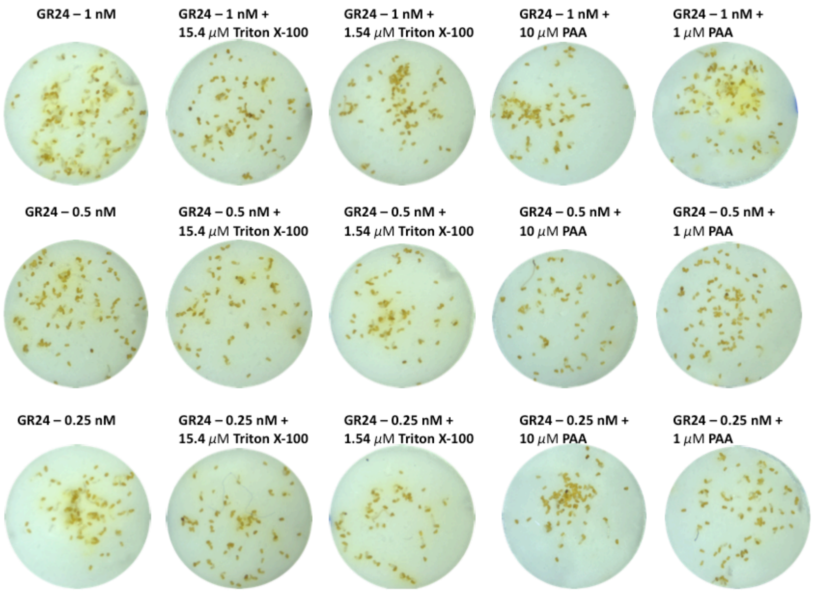

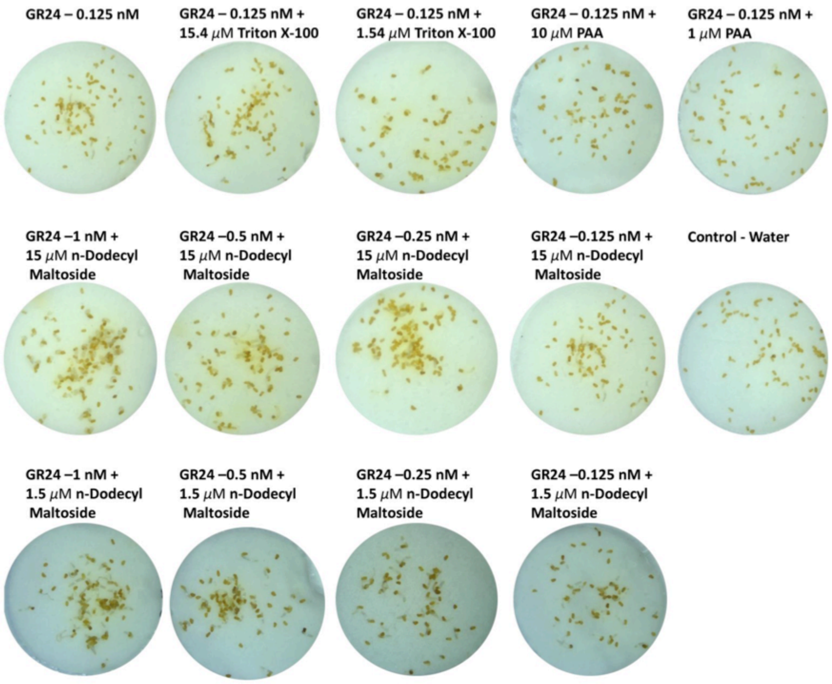

Appendix Figure S8. ***Striga*** **germination assay.**

Representative images of the plates used for *Striga* germination assay.

Appendix Table S1. Primers sequences used for protein cloning

**Appendix Table S1. Primers sequences used for protein cloning**		
Primer name	Sequence (5'-3')	Restriction site
ShHTL7-F	CGggatccATGAGCTCAATTGGATTAGCCC	*Bam*HI
ShHTL7-R	CCGctcgagTCAGTGATCCGTGATGTCCTG	*Xho*I
AtD14-F	CGgaattcATGAGTCAACACAACATCTTAGAA G	*Eco*RI
AtD14-R	ACGCgtcgacTCACCGAGGAAGAGCTCG	*Sal*I
OsD14-F	CGggatccATGCTGCGATCGACGCATCC	*Bam*HI
OsD14-R	CCGctcgagTTAGTACCGGGCGAGAGCGC	*Xho*I
ShHTL7-S95C-F	CGggatccATGAGCTCAATTGGATTAGCCC	*Bam*HI
ShHTL7-S95C-R	CCGctcgagTCAGTGATCCGTGATGTCCTG	*Xho*I
ShHTL7-L143Y-F	GAGCAGAAGGTGATGGATGAGACGTACAG GTCCTTGGACGAGAAC	
ShHTL7-L143Y-R	GTTCTCGTCCAAGGACCTGTACGTCTCATC CATCACCTTCTGCTC	

Appendix Text S1:

Predicting Triton binding by ShHTL family members

To evaluate the capacity of Triton to bind to ShHTLs other than ShHTL7, we produced homology models for all 11 *Striga* HTL proteins, using the crystal structures of ShHTL5 and Triton-bound ShHTL7 as template, and inspected the models to estimate the capacity for binding to Triton. This capacity was evaluated based on the model's active site pocket shape and residue composition, and on the capacity of helix α3 to adopt the open position found in Triton-bound ShHTL7. Conclusions are as follows:

ShHTL1: Triton binding is unlikely because T142L, L153W and T157F cause steric clashes. L143F might preclude the open helix α3 position.

ShHTL2: Triton binding is unlikely because T142L, L153W, T157F, T190F and C195F cause steric clashes. Moreover, the residues of α3 that contact α6 are all short-chained residues, and hence are expected to lead to a different positioning of α3 on α6. M219A deletes an important hydrophobic contact and adds an opening in the binding site that would not be filled by Triton.

ShHTL3: Triton binding is unlikely because T142L, L153W, T157F, T190F, C195F cause steric clashes. The residues of helix3 that contact helix 6 are all short-chained residues, and hence are expected to lead to a positioning of α3 on α6 different from that needed for Triton binding. M219A deletes an important hydrophobic contact and adds an opening in the binding site that would not be filled by Triton.

ShHTL4: Triton binding cannot be excluded, but affinity might be lost substantially because of T142M, L146M, L153M and M219L.

ShHTL5: Triton binding is unlikely because T142L and T157Y cause steric clashes, L143Y may preclude open helix3 positioning. L153M might be suboptimal.

ShHTL6: Triton binding is unlikely because T142L, T157Y and C194H cause steric clashes, and the polar nature of C194H is unfavorable in this position which is in close contact with the hydrophobic moiety of Triton. M219A deletes an important hydrophobic contact and adds an opening in the binding site that would not be filled by Triton.

ShHTL8: Triton binding cannot be excluded, but affinity might be lost substantially because C194F makes the pocket smaller and L153M is suboptimal for binding.

ShHTL9: Triton binding cannot be excluded, but affinity might be lost substantially because T142L, T157Y causes steric clashes and L153M is suboptimal for binding.

ShHTL10: Triton binding is unlikely because T142I, L153F and C194Y cause steric clashes; M219S deletes an important hydrophobic contact and adds an opening in the binding site that would not be filled by Triton.

ShHTL11: Triton binding is unlikely because T142M, L153F, C194F and S154Y cause steric clashes; M219V and Y26A delete important hydrophobic contacts and add openings in the binding site that would not be filled by Triton.
