# Supplementary material for: Structural basis for specific inhibition of the highly sensitive ShHTL7 receptor
Source: EMBO Rep. 2018 Jul 18;19(9):e45619. doi: 10.15252/embr.201745619 (PMC6123649; doi:10.15252/embr.201745619)
Supplement: Supplementary file 2 — Expanded View Figures PDF [file EMBR-19-e45619-s002.pdf]

Expanded View Figures

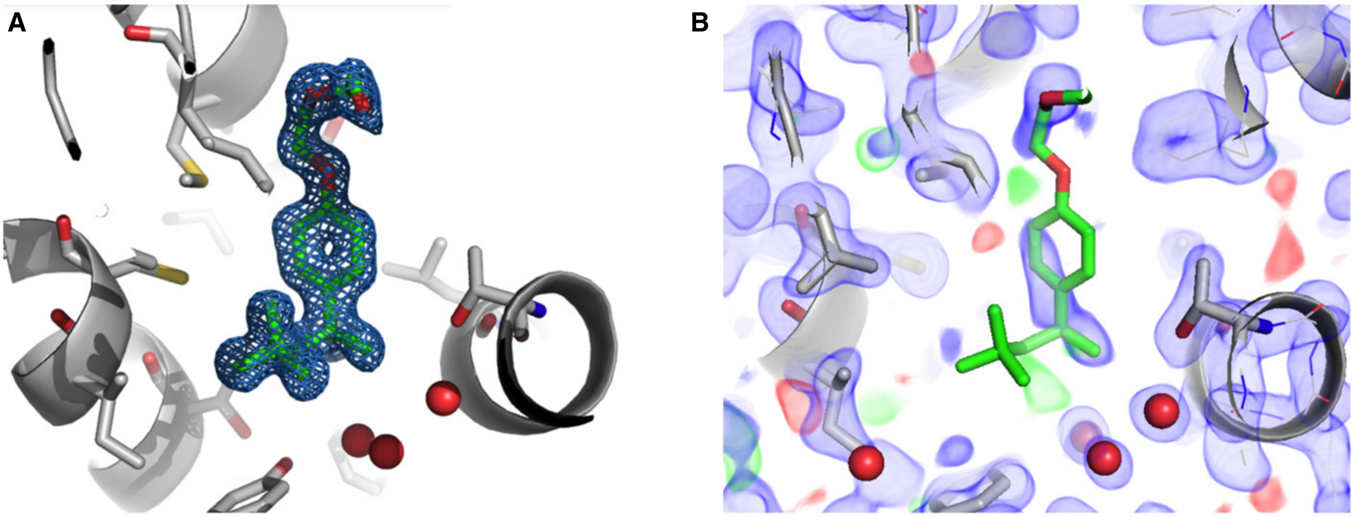

Figure EV1. Structure and affinity of Triton (related to Fig 1A–D).

A 2FoFc omit map (blue) of the active site pocket of ShHTL7$_{S95C}$ (gray) contoured at 1σ cutoff. The final Triton model is shown as stick model, with carbons in green and oxygens in red. The bias of Triton was removed prior to map calculations by 200 cycles of MD simulations followed by refinement of the structure without Triton. Red spheres are water molecules.

B Absence of Triton electron density in "true" ShHTL7-apo structures, illustrated by view of the binding pocket. The 2FoFc map (blue) was contoured at 1σ cutoff. For illustrative purposes, Triton (green and red stick model) from the ShHTL7-Triton complex structure is shown in its position in the binding pocket.

© 2018 The Authors

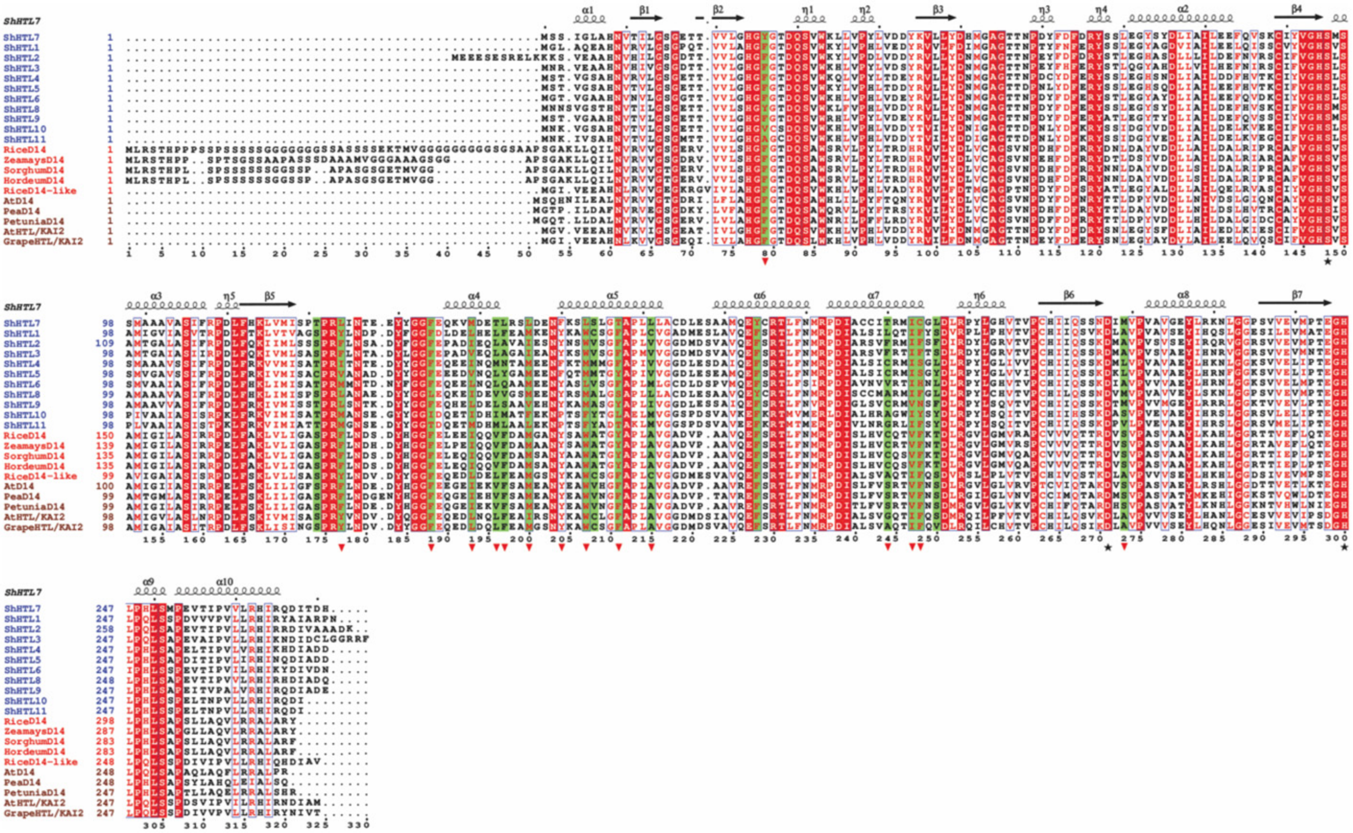

Figure EV2. Sequence alignment of ShHTLs and non-parasitic homologs (related to Fig 1E).

At: *Arabidopsis thaliana*. red background: strict identity; red letter: similar physiological character; blue box: highly conserved blocks; red triangle: amino acids involved in Triton binding. Green background: residues forming the active site pocket. Black star: residues of the catalytic triad.

 © 2018 The Authors

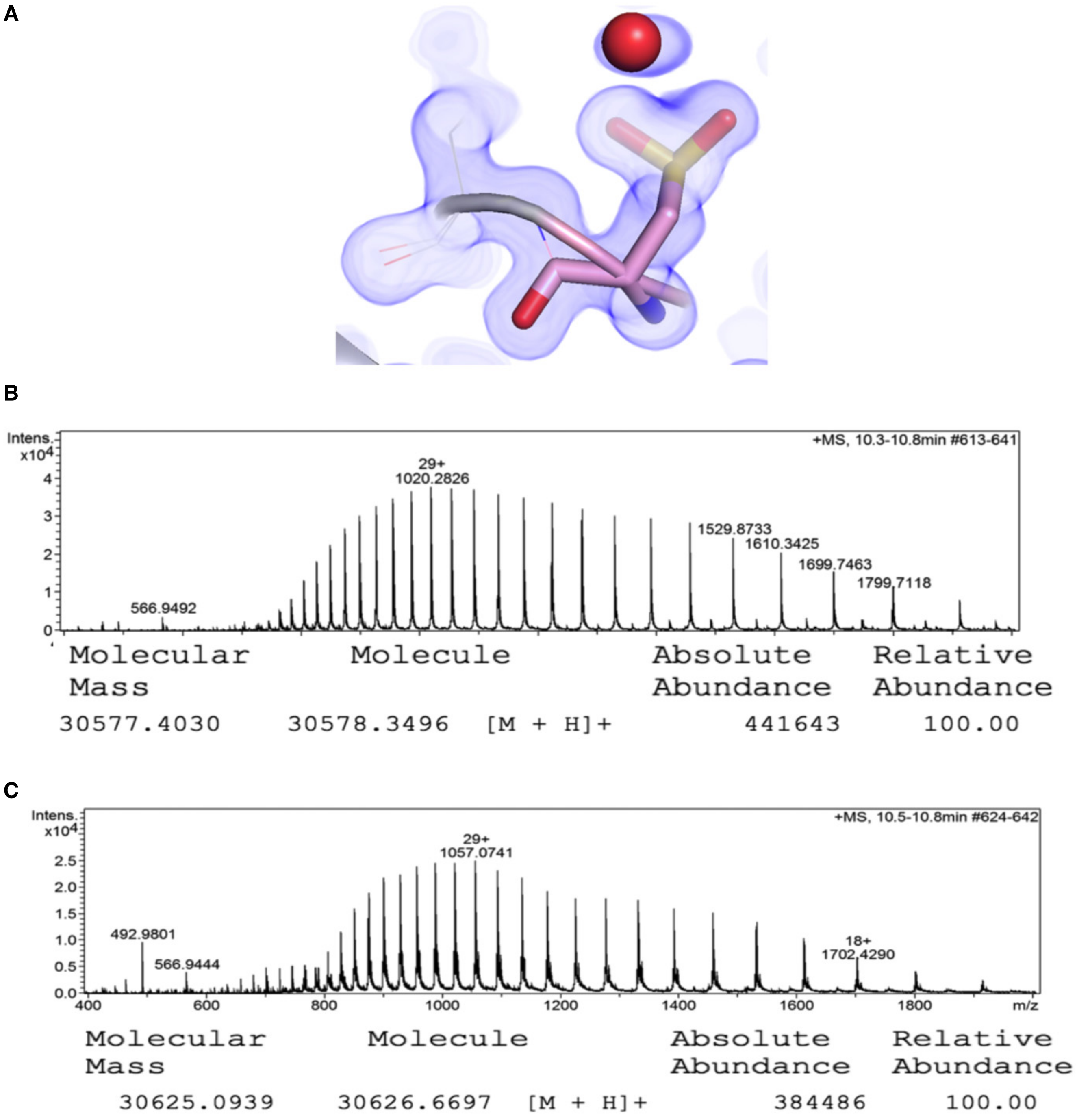

Figure EV3. C95 of the ShHTL7$_{S95C}$ active site mutant is converted to sulfinic acid (related to Fig 1).

A 1σ cutoff 2FoFc map (blue) of C95. Red sphere: water molecule.

B, C Mass spectrometric determination of the molecular weight of (B) wild-type ShHTL7 (calculated Mw = 30,580 Da), and of (C) ShHTL7$_{S95C}$ (calculated Mw with sulfinic acid = 30,628 Da, and without = 30,596 Da).

© 2018 The Authors

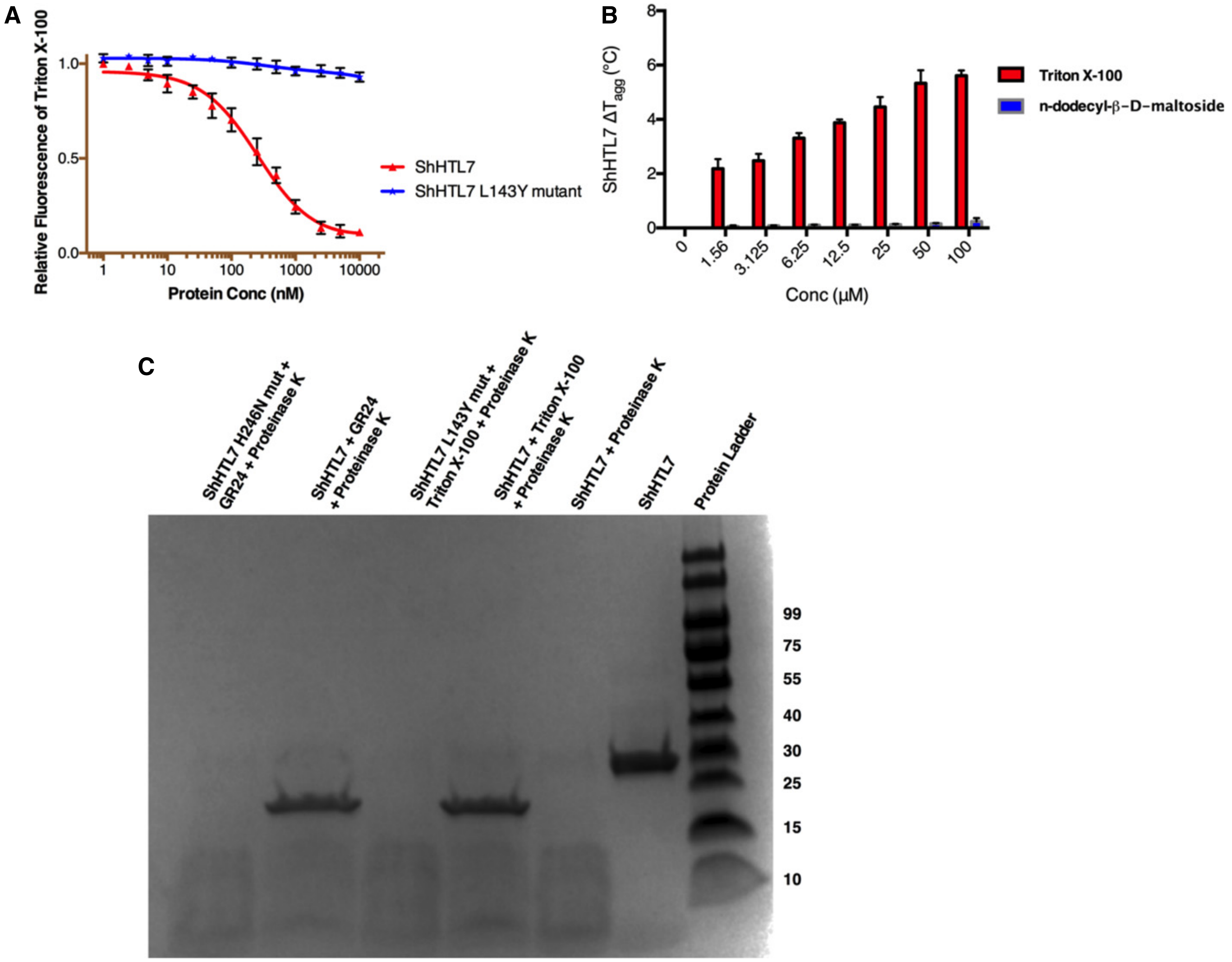

Figure EV4. ***In vitro*** **interaction studies (related to Fig 2).**

A Triton fluorescence in the presence of ShHTL7 and its L143Y mutant without Tween-20 in the buffer. Data are mean ± SD, $n = 3$.

B The change in ShHTL7 aggregation temperature (ΔT_{agg}) plotted against Triton and n-dodecyl-β-D-maltoside concentrations. Data are means ± SD, $n = 3$.

C DARTS assay probing the effect of Triton and GR24 on the stability of ShHTL7 when treated with proteinase K. The ShHTL7 mutant H246N is designed to knock out the catalytic histidine required for SL binding. The L143Y mutant, located in helix α4, precludes Triton binding as shown in (A).

© 2018 The Authors

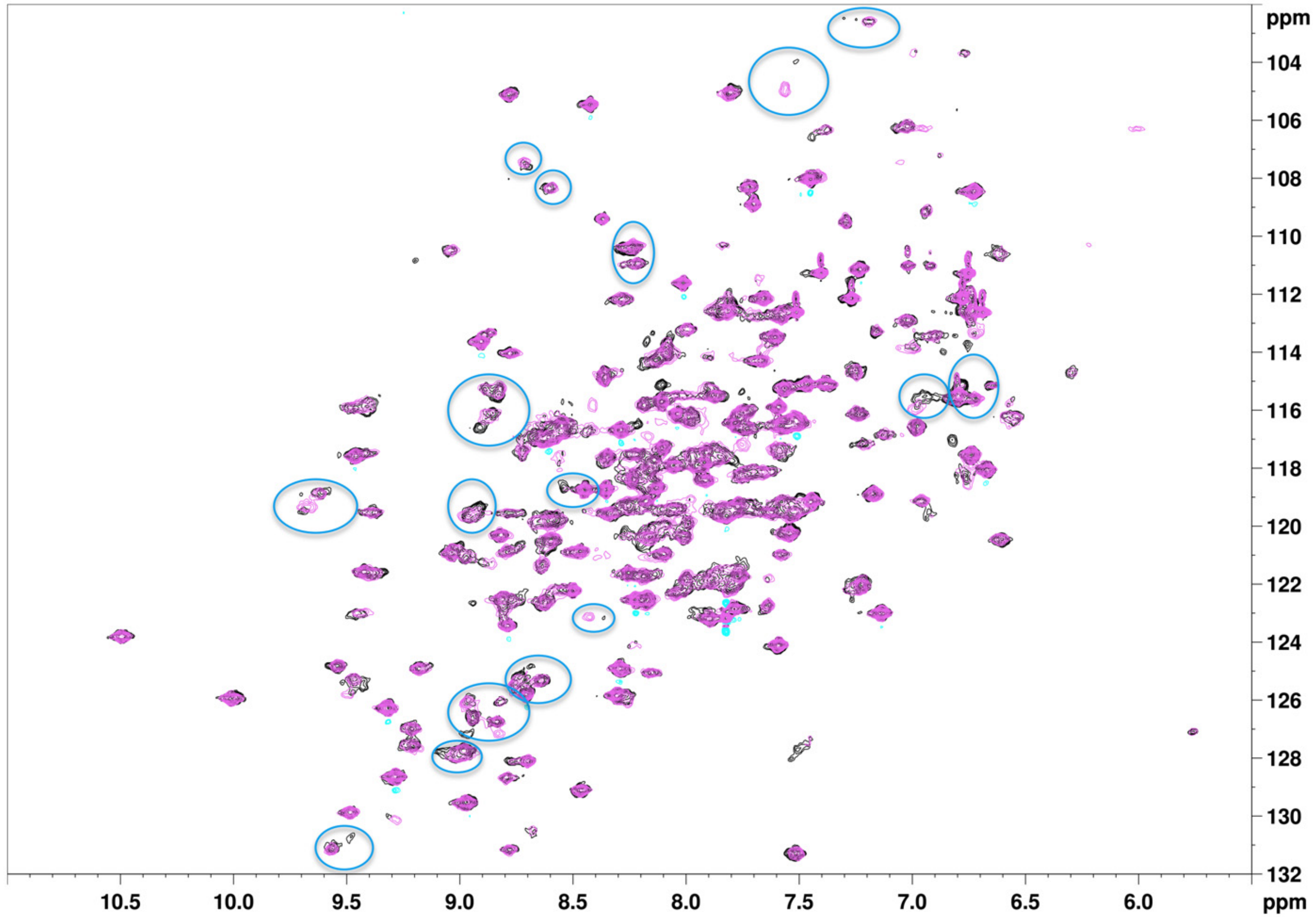

Figure EV5. The presence of Triton induces chemical shifts changes in ^{15}N-ShHTL7 (related to Fig 3A).
Superimposed are NMR HSQC measurements of ^{15}N-ShHTL7 purified without Triton (gray) and purified in the presence of Triton (pink). The blue circles highlight prominent changes in the position and/or intensity of several well-dispersed chemical shifts.

© 2018 The Authors
